# Xeroderma pigmentosum et Dermoscopie

**DOI:** 10.11604/pamj.2013.16.105.3219

**Published:** 2013-11-18

**Authors:** Kawtar Inani, Fatimazahra Mernissi

**Affiliations:** 1 Service de Dermatologie, CHU Hassan II, Fès, Maroc

**Keywords:** Xeroderma pigmentosum, dermoscopie, génodermatose info, Xeroderma pigmentosum, dermoscopy, genodermatosis

## Image en medicine

Le Xeroderma pigmentosum (XP) est une génodermatose rare, fréquente au Maghreb vu le taux élevé de mariage consanguin. Ses manifestations cutanées sont dominées par la fréquence de cancers cutanés. La dermoscopie est une méthode non invasive, qui facilite l'orientation diagnostique et la surveillance de ces malades permettant ainsi de réduire le recours à une chirurgie délabrente en cas de diagnostic tardif de ces tumeurs. En effet, sur un terrain d'XP, les lésions pigmentées provoquées par les UV, l'état poikilodermique, et la fréquence des kératoses actiniques, rendent le diagnostic clinique difficile. Une fillette de 11 ans, avec notion de consanguinité et deux cas similaire dans la famille, s'est présentée pour prise en charge de lésions papuleuses du visage. L'examen clinique a trouvé une poikilodermie des zones photo-exposées, une tumeur ulcérée au niveau de la paupière inférieure droite, évocatrice d'un carcinome épidermoide (CE) ou d'un carcinome baso-cellulaire (CBC), des papules noirâtres évocatrices de CBC. La dermoscopie a permis de réconforter notre approche clinique, en objectivant pour la tumeur de la paupière, une vascularisation polymorphe en faveur d'un CE. Pour les lésions papuleuses, une vascularisation en tronc d'arbre, des nids ovoïdes ainsi qu'un voile bleu gris en faveur d'un CBC. Un traitement par photothérapie dynamique a été proposé pour les CBC de petite taille, une chirurgie pour la tumeur de la paupière, ainsi d'une surveillance régulière afin de détecter de nouvelles lésions.

**Figure 1:**
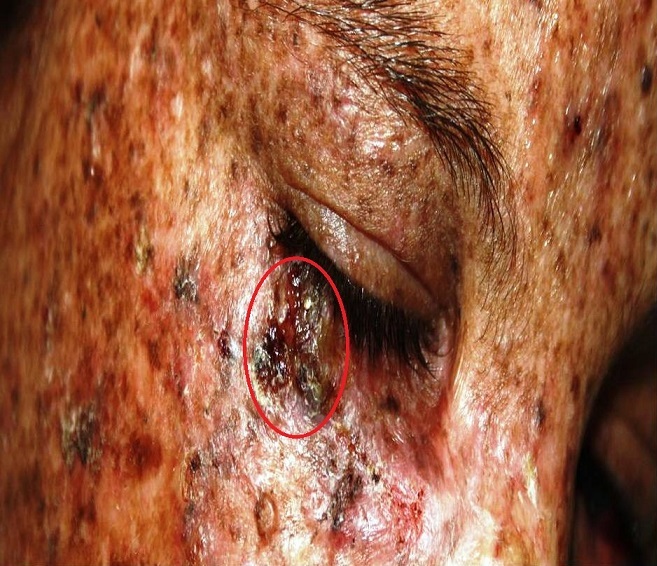
Tumeur ulcérée de la paupière inférieure droite; papules noirâtres au niveau du front; vascularisation linéaire irrégulière et en points

